# Probiotics, prebiotics, and synbiotics supplementation in prediabetes: protocol for a systematic review and meta-analysis

**DOI:** 10.1097/MD.0000000000019708

**Published:** 2020-03-27

**Authors:** Xuqin Du, Chunguang Xie, Lipeng Shi, Hong Gao, Chan Yang, Qionghui Liu

**Affiliations:** a Hospital of Chengdu University of Traditional Chinese Medicine, Chengdu, Sichuan Province; b Clinical Department, Traditional Chinese medicine hospital Dianjiang Chongqing, Dianjiang, Chongqing, China.

**Keywords:** meta-analysis, prebiotic, prediabetes, probiotic, symbiotic, systematic review

## Abstract

**Background:**

The prevalence of prediabetes and diabetes is increasing rapidly, and 5% to 10% of prediabetic patients will develop diabetes every year. Diabetes causes major health problems as well as a large economic burden. Human studies have demonstrated the beneficial effects of probiotics, prebiotics, and synbiotics supplementation in prediabetes. However, there are no systematic reviews that explore the therapeutic efficacy of probiotics, prebiotics, and synbiotics supplementation in patients with prediabetes. Therefore, we aim to synthesize the existing evidence evaluating the effectiveness and safety of probiotics, prebiotics, and synbiotics supplementation in prediabetic patients.

**Methods:**

We will search PubMed, EMBASE, Cochrane Library, Web of Science, the Clinical Trials.gov website, China National Knowledge Infrastructure, China Science and Technology Journal Database, and Wanfang Data Knowledge Service Platform from inception to August 2020. Additionally, the search will be conducted in multiple languages. Search terms are keywords and medical subject headings related to prediabetes, probiotics, prebiotics, and synbiotics. The primary outcomes are differences in glycated hemoglobin and fasting blood glucose. The secondary outcomes are differences in fasting insulin, homeostasis model assessment of insulin resistance, quantitative insulin sensitivity check index, and adverse events. The meta-analysis will be performed using the Revman5.3.0 software provided by the Cochrane Collaboration.

**Results:**

Our study will systematically evaluate the effectiveness and safety of probiotics, prebiotics, and synbiotics supplementation in prediabetes.

**Conclusion:**

The findings of this study will provide the best available evidence for probiotics, prebiotics, and synbiotics in the treatment of prediabetes, and provide a strong basis for clinical treatment.

## 1 Introduction

Prediabetes, typically defined as glycaemic parameters above normal levels but below diabetes thresholds, is a metabolic state between normal blood glucose and diabetes, including impaired fasting glucose, impaired glucose tolerance, and impaired fasting glucose combined with impaired glucose tolerance.^[1]^ With the socio-economic development and the change of human diet structure, the prevalence of diabetes, and prediabetes is increasing rapidly. The number of diabetic patients worldwide is projected to rise from 382 million in 2013 to 592 million in 2035.^[2]^ It is estimated that 470 million people worldwide will suffer from prediabetes by 2030, and 5% to 10% of prediabetic patients will develop diabetes every year.^[1]^ Diabetes causes major health problems as well as a large economic burden. The direct annual cost of diabetes worldwide exceeds US$827 million.^[3]^ Moreover, diabetics lose a great deal of labor value due to reduced productivity and production time. Consequently, timely and effective preventive measures in prediabetes are a reasonable way to prevent the diabetes epidemic and lessen the healthcare cost.

One study has shown that early intervention in prediabetes can reduce the risk of developing type 2 diabetes by 58%.^[4]^ Effective interventions conclude lifestyle changes and medications.^[5–7]^ However, in clinical practice, the adherence to lifestyle changes in prediabetic populations is low, and the compliance of using hypoglycemic drugs is relatively poor. Thus, to find a nonhypoglycemic agent is of great significance in preventing the conversion of prediabetes to diabetes. Recent studies indicate that alterations in gut microbiota play a major role in the pathogenesis of diabetes.^[8–10]^ It is reported that when the intestinal dysbacteriosis, the production of lipopolysaccharide increases, the release of pro-inflammatory cytokines increases, and a series of nonspecific inflammatory responses occur in the body, which interferes with insulin signal transduction, induce insulin resistance and lead to hyperinsulinemia,^[11]^ as well as trigger chronic low-level inflammatory responses of islet cells and metabolic endotoxemia, and ultimately, lead to destruction and apoptosis of islet β cells.^[12,13]^ Microecological preparations, including probiotics, prebiotics, and synbiotics, have beneficial health effects on the host when administered in sufficient amounts. Studies suggest that certain microecological preparations can exert anti-diabetic effects in different studies,^[14,15]^ improving glycemia, insulin sensitivity and inflammatory markers in subjects with type 2 diabetes.^[16–19]^ Several human studies have also demonstrated the beneficial effects of microecological preparation supplementation in prediabetes.^[20,21]^

To the best of our knowledge, up to date, there are no systematic reviews that explore the therapeutic efficacy of probiotics, prebiotics, and synbiotics supplementation in patients with prediabetes. The aim of this systemic review and meta-analysis is to synthesize the existing evidence evaluating the effectiveness and safety of probiotics, prebiotics, and synbiotics supplementation in prediabetic patients.

## 2 Methods

### 2.1 Eligibility criteria

#### 2.1.1 Types of trials

Only randomized controlled trials (RCTs).

#### 2.1.2 Participants

All adult patients who met the accepted diagnostic criteria for prediabetes will be included in this study without discrimination of race or gender.

#### 2.1.3 Types of interventions

Only RCTs comparing probiotics, prebiotics, and synbiotics supplementation with placebo will be included in this study. Also, the minimum duration of treatment is 8 weeks.

#### 2.1.4 Types of controls

RCTs with placebo treatment for at least 8 weeks will be included as the controls in this study.

#### 2.1.5 Outcomes

##### 2.1.5.1 Primary outcomes

(1) Differences in glycated hemoglobin;
(2) Differences in fasting blood glucose.

##### 2.1.5.2 Secondary outcomes

(1) Differences in fasting insulin;
(2) Differences in homeostasis model assessment of insulin resistance;
(3) Differences in quantitative insulin sensitivity check index;
(4) Incidence and severity of adverse events.

### 2.2 Data sources and selection strategy

We will utilize the computer to retrieve the following databases: PubMed, EMBASE, Cochrane Library, Web of Science, the Clinical Trials.gov website, China National Knowledge Infrastructure, China Science and Technology Journal Database, and Wanfang Database from inception to August 2020. Additionally, the search will be conducted in multiple languages. Search terms are keywords and medical subject headings related to prediabetes, probiotics, prebiotics, and synbiotics. Keywords and medical subject headings used in this study include: “prediabetic state,” “prediabetes,” “glucose intolerance,” “impaired glucose tolerance,” “impaired fasting glucose,” “probiotics,” “prebiotics,” “synbiotics,” “randomized controlled trials,” and “clinical trial.” The search strategy will be determined by 2 researchers after several pre-searches. The PubMed search strategy is outlined in Table 1.

**Table 1:**
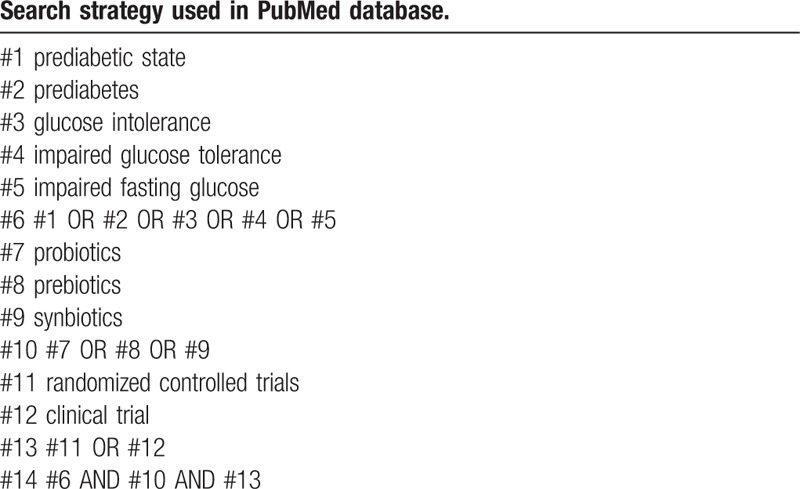
The PubMed search strategy.

### 2.3 Data selection

**Figure 1:**
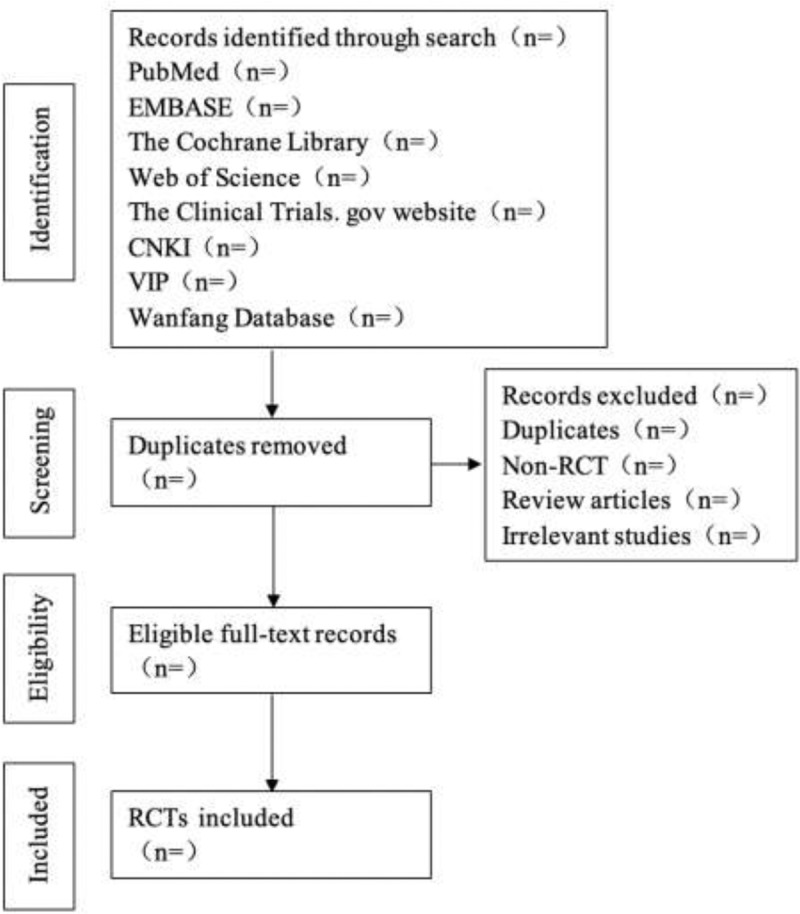
Flow chart of the study selection.

The literature included in the meta-analysis will be independently screened by 2 reviewers according to the eligibility criteria listed in this study. In the process of literature selection, the title and abstract will be read first, and the full text will be further read to determine the final inclusion after excluding the obvious irrelevant literature. The reporting quality of the included studies will be independently assessed by 2 reviewers using the PRISMA evaluation scale.^[22]^ Furthermore, the meta-analysis protocol of this study was created according to the PRISMA-P guidelines.^[23]^ The process of literature identification and screening is shown in Figure 1.

### 2.4 Data extraction

According to the pre-designed data extraction table, data will be extracted independently by 2 reviewers. All studies that meet the eligibility criteria will be extracted as follows:

(1) Basic information, including study design, author, country, publication year source of literature, sample size, and grouping;
(2) Basic characteristics, including age, gender, and the number of participants;
(3) Methodological description, including randomized method, blind method, allocation concealment, losses to follow-up, and assessed risk of bias;
(4) Interventions and controls characteristics, including time and type of interventions and controls;
(5) Study outcomes, including primary and secondary outcome indicators, as well as adverse events.

During the data extraction process, the extracted data will be checked by a third reviewer for verification. Moreover, if there is any disagreement in the process of data extraction, it needs to be re-examined and discussed, and even the judgment of a third reviewer.

### 2.5 Missing data management

We will give a call or sent an e-mail to the author to obtain relevant data. The study will be deleted if the missing data is unavailable. Besides, we will perform a sensitivity analysis of the missing data and evaluate its impact on this study.

### 2.6 Risk of bias

The quality of the study will be assessed by 2 reviewers in accordance with the “biased risk assessment” Handbook 5.3 provided by the Cochrane Collaboration. There are 7 main items:

(1) Random allocation method;
(2) The allocation scheme is hidden;
(3) Blind methods are used for subjects and treatment planners;
(4) Blind method for measurement results;
(5) The integrity of the resulting data;
(6) Selectively report the results of the study;
(7) Other sources of bias.

Finally, the “low risk of bias,” “high risk of bias,” and “uncertain risk of bias” of the study will be judged. If the opinions are inconsistent, they will be resolved through group discussion.

### 2.7 Statistical analysis

The meta-analysis will be performed using the Revman5.3.0 software provided by the Cochrane Collaboration. The relative risk and the 95% confidence intervals will be used for the dichotomous variables. And the mean difference or standard mean difference will be used as a therapeutic statistic for the continuous variables, along with 95% confidence intervals. The heterogeneity test utilizes the *I*^2^ statistical value. When there is statistical homogeneity between the studies (*I*^2^ ≤ 50% or *P > *.10), a fixed effect model will be employed to assess the difference. Conversely, when there is statistical heterogeneity between the studies (*I*^2^ ≥ 50% or *P < *.10), the evaluator will conservatively assess the difference using a random effects model. Besides, the results of individual studies will be summarized descriptively if fewer studies are unable to carry out a meta-analysis.

### 2.8 Additional analysis

#### 2.8.1 Publication bias

A funnel plot analysis will be performed on the indicators with the largest number of literatures (≥10 articles) to detect the possibility of publication bias.

#### 2.8.2 Subgroup analyses

We will perform a subgroup analysis of the different dosage and duration of interventions, respectively.

#### 2.8.3 Sensitivity analyses

To ensure the stability of the results, we will remove each study in turn for sensitivity analysis to assess whether the deleted study affects the overall effect.

#### 2.8.4 Quality of evidence

After completing the data extraction and evaluation analysis, we will use the GRADE pro 3.2 software to assess the overall quality of the evidence and the strength of the recommendation.^[24]^

## 3 Discussion

The increasing number of research on probiotics, prebiotics, and synbiotics for prediabetes leads us to believe that these interventions have improved glycemia and insulin sensitivity. The purpose of this systemic review and meta-analysis is to objectively estimate the effects of probiotics, prebiotics, and synbiotics supplementation on glycemic control and insulin metabolism in prediabetic patients. To interpret the results accurately, we will ascertain the influence of efficacy and safety in different dosages of probiotics, prebiotics, and synbiotics and different duration of treatment. The strength of this systematic review is that screening, data extraction, and quality assessment will be performed independently by 2 reviewers. Herein, this systematic review and meta-analysis will be the first one, which employ rigorous methods to identify and collate the best available evidence assessing the effectiveness and safety of probiotics, prebiotics, and synbiotics supplementation in prediabetes, therefore informing clinical treatment decisions.

## Author contributions

**Conceptualization:** Xuqin Du, Chunguang Xie, Lipeng Shi.

**Data curation:** Xuqin Du.

**Funding acquisition:** Qionghui Liu.

**Investigation:** Xuqin Du.

**Methodology:** Lipeng Shi, Xuqin Du.

**Project administration:** Lipeng Shi, Xuqin Du.

**Software:** Lipeng Shi.

**Supervision:** Chan Yang.

**Validation:** Hong Gao.

**Writing – original draft:** Lipeng Shi, Xuqin Du.

**Writing – review and editing:** Lipeng Shi, Xuqin Du.

Lipeng Shi orcid: 0000-0002-4478-0622.
